# Mayaro Virus: The Potential Role of Microbiota and *Wolbachia*

**DOI:** 10.3390/pathogens10050525

**Published:** 2021-04-27

**Authors:** Thiago Nunes Pereira, Fabiano Duarte Carvalho, Jerônimo Nunes Rugani, Vanessa Rafaela de Carvalho, Jaqueline Jarusevicius, Jayme A. Souza-Neto, Luciano Andrade Moreira

**Affiliations:** 1 Grupo Mosquitos Vetores: Endossimbiontes e Interação Patógeno-Vetor, Instituto René Rachou-Fiocruz, Belo Horizonte 30190-002, Brazil; thiagonp@hotmail.com (T.N.P.); fabiano.carvalho@fiocruz.br (F.D.C.); 2 Grupo Taxonomia de Flebotomíneos e Epidemiologia das Leishmanioses, Instituto René Rachou-Fiocruz, Belo Horizonte 30190-002, Brazil; jeronimomnr@hotmail.com; 3 Department of Bioprocesses and Biotechnology, School of Agricultural Sciences, São Paulo State University (UNESP), Botucatu 18610-034, Brazil; vanessa.carvalho@unesp.br (V.R.d.C.); jayme.souza-neto@unesp.br (J.A.S.-N.); 4 School of Agricultural Sciences, Central Multiuser Laboratory, São Paulo State University (UNESP), Botucatu 18610-034, Brazil; 5 Institute of Biotechnology, São Paulo State University (UNESP), Botucatu 18607-440, Brazil; jaquejaru@yahoo.com.br

**Keywords:** Mayaro virus, *Aedes albopictus*, vector competence, microbiota and *Wolbachia*

## Abstract

The Mayaro virus (MAYV) is an arbovirus that circulates mainly in tropical forests or rural areas in Latin America and is transmitted mainly by *Haemagogus* mosquitoes. The objective of this study was to evaluate the vector competence, microbiome, and the presence of *Wolbachia* in three *Aedes albopictus* populations infected with MAYV. The vector competence was assessed based on viral infection and transmission by RT-qPCR. In addition, the microbiome was evaluated by amplification of the 16S rRNA V4 region and PCR to detect the presence of *Wolbachia* (strain *w*AlbA/*w*AlbB). Our results show that all three populations were susceptible to MAYV infection. The potential transmission of the MAYV was consistent in all populations of naïve mosquitoes injected (more than 50%). The microbiome analysis revealed 118 OTUs (operational taxonomic unit) from the three populations, 8 phyla, 15 classes, 26 orders, 35 families, 65 genera, and 53 species. All populations had *Pseudomonas* and *Wolbachia* as predominant genera. There was no difference between the variables for MAYV and *Wolbachia* (*w*AlbA or *w*AlbB) in the abdomen. However, in the head + thorax samples at 14 dpi, there was a difference between the two populations, indicating a possible correlation between the presence of *Wolbachia* (*w*AlbB) and infection. Overall, we show evidence that *Ae. albopictus* displays significant infection and transmission competence for the MAYV in the laboratory, and its bacterial microbiota play an important role in the host, mainly the strains of *Wolbachia*. The influence of the intestinal microbiota of *Ae. albopictus* is poorly known, and a better understanding of these interactions would open new perspectives for disease control through the manipulation of microbial communities. The exact contribution of this mosquito species to the transmission of the MAYV in the field remains to be confirmed.

## 1. Introduction

Vector-borne diseases are among the main causes of human morbidity and mortality. Every year, more than one billion people are infected and more than one million people die from vector-borne diseases [1]. Annually, these diseases account for significant impacts to public health services and negatively impact development and economic growth [2].

The mosquito *Aedes albopictus* is native to the forests of Southeast Asia, where it is an important vector for different arboviruses, posing a potential risk to public health [3,4,5]. This invasive mosquito species was first recorded in Brazil in 1986 in the state of Rio de Janeiro [6]. Current data shows that *Ae. albopictus* is present in 25 out of 27 Brazilian states [7,8]. 

For a long time, *Ae. albopictus* was considered by many authors as a species of relatively low importance in the transmission of pathogens to humans, minimizing their importance in public health issues. However, over the past years, many studies have demonstrated the vector competence of *Ae. albopictus* for arboviruses [9,10,11,12,13].

Important vector-borne diseases have re-emerged or spread to new parts of the world, and an example is the Mayaro virus (MAYV). This virus is an *Alphavirus*, member of the *Togaviridae* family, and is closely related to the Chikungunya virus (CHIKV) [14], with both displaying similar clinical symptoms in humans, including high fever, frontal headache, maculopapular eruption, photophobia, nausea, epigastric pain, myalgia, and severe arthralgia, which may persist for months or even years [15,16]. Given the high degree of similarity in symptoms caused by arboviruses, it is difficult to distinguish and diagnose the causative agents of infections in humans; this can ultimately lead to underestimation of the real prevalence and circulation of viruses, such as the MAYV, in the population [17,18]. 

Several cases of the MAYV have occurred in different localities in Brazil, including the Northern, Northeastern, and Central West regions, with frequent occurrence in the states of Pará, Amazonas, Acre, and Mato Grosso [17,19,20,21,22,23,24]. Such notifications indicate the circulation of the MAYV and consequently reinforce the importance of mosquito vector competence studies. 

The relationship between microbiota and vector competence is not fully understood; however, it is known that the natural bacterial microbiota has been shown to contribute to immunity, vector competence, and influence pathogen development [25,26], as well as nutritional aspects [27]. Regarding the bacterial composition, a very important relationship with the endosymbiotic bacterium called *Wolbachia pipientis* needs mentioning. *Wolbachia* is a maternally inherited alphaproteobacterium estimated to infect up to 60% of arthropod species [28,29]. This bacterium is a promising biocontrol agent due to its ability to modulate replication of some arboviruses in *Ae. Aegypti* [30,31,32]. The mosquitoes *Ae. albopictus* are naturally infected with the *Wolbachia* strains *w*AlbA and/or *w*AlbB [33,34], which in some cases can modulate the vector competence of its hosts.

Thus, considering the contribution of microbiota in vector competence and the few studies related to the vector competence of *Ae. albopictus* for the MAYV, we have shown that *Ae. albopictus* of distinct field-derived populations display significant infection and transmission competence for the MAYV. Additionally, it was observed that the bacterial microbiota play an important role in the host, mainly when the strains of *Wolbachia* are present.

## 2. Materials and Methods

### 2.1. Mosquito Rearing

*Ae. albopictus* eggs were collected through ovitraps from the following states in Brazil: Minas Gerais (Belo Horizonte City/19°52′12.4″ S 43°58′14.5″ W), Rio de Janeiro (Rio de Janeiro City/22°52′39.2″ S 43°14′25.4″ W), and Santa Catarina (Tubarão City/28°28′40.0″ S 49°01′25.0″ W). All collections occurred in March 2017. The eggs were hatched at the Instituto René Rachou, Fiocruz/MG in a controlled insectary environment at 27 ± 2 °C, ∼82% RH and 12 h light/dark regime. Larvae were fed with TetraMin Tropical (Tetra^®^fish food, Melle, Germany) ad libitum, and adults were given 10% sucrose solution ad libitum. Adult mosquitoes were visually screened to select only *Ae. albopictus*. Adult females were fed blood from a human blood bank (see mosquito infection) using an artificial water-jacketed membrane feeding apparatus for egg production. All experiments were performed using females from a colony derived from within no more than two generations of egg collection. 

### 2.2. Virus

The MAYV was maintained in the *Ae. albopictus* cell line (C6/36) in Leibowitz L-15 medium supplemented with 10% foetal calf serum at 28 °C. On the first mosquito infection replicate (A), the viral supernatant was harvested at 5 days after cell infection, with a viral titer of 1 × 10^9^ PFU/mL, and for the second mosquito infection replicate (B), it was harvested at 4 days after cell infection, with a viral titer of 6 × 10^9^ PFU/mL. Both viral batches were quantified after a freezing process. 

### 2.3. Mosquito Infection

Through an artificial water-jacketed membrane feeding apparatus set up at 38 °C, 5-day-old mosquitoes were challenged with an infectious blood meal spiked with a cultured viral supernatant (two virus: one blood) for 45 min. The human blood used in all experiments was obtained from a blood bank (Fundação Hemominas, Belo Horizonte, MG, Brazil), according to the terms of an agreement with Instituto René Rachou, Fiocruz/MG (OF.GPO/CCO agreement-Nr 224/16). Fully engorged females were separated on a cold Petri dish and maintained in cages with 10% sucrose solution ad libitum until collection. Whole mosquitoes were collected on different days post-infection (dpi) and stored at −80 °C. 

### 2.4. Saliva Collection

Fourteen dpi, mosquitoes were anaesthetized by CO_2_ exposure and kept on an ice plate while the wings and legs were removed. Each mosquito had its proboscis inserted into a 10 μL pipette tip containing 10 μL of a 1:1 solution of 30% sucrose and sterile fetal bovine serum for the salivation process [35]. After 30 min, the contents of the tips were individually collected and stored at −80 °C.

### 2.5. Saliva Nanoinjection

Eight samples of undiluted saliva from each *Ae. albopictus* population were individually injected (207 nL dose) into the pleural membranes of 15 naïve *Ae. aegypti* using the Nanoject II injector (Drummond Sci, Broomall, PA, USA). Five days after, whole nanoinjected mosquitoes were collected, and viral presence was determined by RT-qPCR analysis on an average of six mosquitoes per group. We used *Ae. aegypti* mosquitoes for saliva nanoinjection given their confirmation as good amplification hosts for this virus [13].

### 2.6. RNA Extraction and Real-Time RT-qPCR

Total RNA/DNA was extracted from the head + thorax of each individual female mosquito using the High Pure Viral Nucleic Acid Kit (Roche), following manufacturer’s instructions. A specific set of primers and probes were used for the MAYV [36] and RPS17 [37].

To assess the presence of the MAYV (mosquito infection), total head + thorax RNA was used, whereas for transmission potential, the RNA was extracted from whole saliva-injected mosquitoes. RNA samples were quantified using the NanoDrop^TM^ 2000 spectrophotometer (Thermo Scientific, Walthman, MA, USA); all samples were diluted to 50 ng/μL.

The thermocycling conditions were as follows: reverse transcription at 50 °C for 10 min; RT inactivation/initial denaturation at 95 °C for 30 s, 40 cycles of 95 °C for 5 s and 60 °C for 30 s, followed by cooling at 37 °C for 30 s. The total reaction volume contained 10 μL (5× LightCycler^®^ Multiplex RNA Virus Master, Roche, Basel, Switzerland), 1 μM primers and probe, and 125 ng of RNA. The MAYV infection levels in mosquitoes were quantified by RT-qPCR using the LightCycler^®^ 96 (Roche, Basel, Switzerland).

The multiplex assay was performed with probe and primers specific for the MAYV: MayV-F/5′/GTG GTC GCA CAG TGA ATC TTTC/3′ and MayV- R/5′/CAA ATG TCC ACC AGG CGA AG/3′ and May-Probe 5’/FAM/ATG GTG GTA GGC TAT CCG ACA GGT C/3lABkFQ/3′ (36). For mosquito detection, the ribosomal gene S17 (RPS17) was used, and the probe and primers were 117S-F 5′/TCC GTG GTA TCT CCA TCA AGC T/3′/e 17S-R 5′/CAC TTC CGG CAC GTA GTT GTC/3′ and probe 5′/HEX/CAG GAG GAG GAA CGT GAG CGC AG/3B [37].

All head + thorax samples were tested in duplicate for the MAYV and analyzed using absolute quantification by serial dilutions of the target gene product (MAYV) cloned into the plasmid pGEMT-Easy (Promega, Madison, WI, USA), according to the manufacturer’s instructions. The head + thorax samples were quantitatively evaluated, whereas whole mosquitoes (after saliva/nanoinjection procedure) were qualitatively evaluated. Negative and positive control samples were normalized and used to determine a threshold between samples.

### 2.7. cDNA Synthesis 

For the assay of identification of *Wolbachia* and metagenomics, after total RNA extraction and quantification via NanoDrop, 1 µg of total RNA was treated with DNAse enzyme (Promega, Madison, WI, USA). Then, the final product (11 µL) was used for cDNA synthesis following the manufacturer’s recommendations, via use of the enzyme M-MLV reverse transcriptase (Promega, Madison, WI, USA). At the end of the process, the cDNA was diluted (1:10) in water free of nucleases and stored at −20 °C.

### 2.8. Amplification of the 16S rRNA V4 Region

To determine the diversity of microbiota, 10 abdomens were used for each population (adult mosquitoes). For the polymerase chain reaction (PCR), primers specific to the hypervariable region V4 [38] for amplification of the 16S rDNA gene were used, according to the methodology used by Caporaso [39]. Each sample was amplified with different forward primers (Supplementary Table S1). The generated libraries were purified with magnetic beads (AMPureAgencourt) and quantified in the QuantStudio 3 (Applied Biosystems) with the KapaSybrFast Universal qPCR Kit. The mean Ct values of each sample were adjusted to a concentration of 4.0 nM.

### 2.9. Illumina Sequencing 

Next generation sequencing (NGS) was performed in the MiSeq Personal Sequencer (Illumina, San Diego, CA, USA) equipment of the Molecular Biology Laboratory, Clinical Hospital fromUNESP School of Medicine (Botucatu São Paulo) by metagenomic workflow with two MiSeq Reagent v2 kits (300 cycles). The data were pre-processed by the MiSeq Reporter software v.10.1.2.

### 2.10. Sequencing Analysis

The software CLC Microbial Genomics Module (Qiagen, Hilden, Germany) was used following the manual guidelines. For the pairing of reads, the parameters used were mismatch cost: 2, minimum score: 8, gap cost: 3, and maximum unalignment and mismatches: 0, followed by setting the lengths of reads. Sample filtering was performed so that all samples had a minimum value of 100 reads and a minimum percentage of 50% away from the median. The operational taxonomic unit (OTU) clustering was done with the SILVA database, with 97% similarity between sequences and filter removal of OTUs of abundance less than 0.005%.

### 2.11. Identification of Wolbachia and Sequencing

Mosquito (abdomen and head + thorax) cDNA (20 ng/μL) were used in PCR reactions for *Wolbachia w*AlbA and *w*AlbB genotyping. The primers *w*AlbA F (5′-GTG TTG GTG CAG CGT ATG TC-3′), *w*AlbA R (5′-GCA CCA GTA GTT TCG CTA TC-3′), *w*AlbB F (5′ACG TTG GTG GTG CAA CAT TTG-3′), and *w*AlbB R (5′-TAA CGA GCA CCA GCA TAA AGC-3′)(34) (Zhou et al., 1998; Armbruster et al., 2003), which amplified a 187 bp and 268 bp fragment, respectively, were used in reactions containing 1.5 mM MgCl_2_, 200 µM dNTP mix (New England Biolabs, Ipswich, MA, USA), 5.0% dimethyl sulfoxide (Invitrogen, Carlsbad, CA, USA), 1.5 units of platinum Taq DNA polymerase (Invitrogen, Carlsbad, CA, USA), 0.4 pmol of sense primer, and 0.4 pmol of antisense primer (IDT Technologies, Coralville, IA, USA). The cDNA amplification was performed in a thermal cycler using 40 cycles of denaturation at 94 °C for 45 s, primer-specific annealing for 45 s (56 °C for *w*AlbA and 59 °C for *w*AlbB) and extension at 72 °C for 45 s, followed by one final extension at 72 °C for 5 min. Amplification products were visualized on 2% agarose gels stained with ethidium bromide (10 µg/mL). Ten samples of each group showing the specific band for both *w*AlbA and *w*AlbB were sequenced by the Fiocruz sequencing platform (Sanger) to confirm species genotyping. 

### 2.12. Data Analysis 

The mosquito infection rate was analyzed with Person omnibus normality and D’Agostino tests. Fisher’s exact test was then used to assess differences in viral prevalence. Comparisons were significant for *p* values lower than 0.05, and viral load data were compared through the Mann–Whitney U test. Pearson’s Chi-squared test was used as an inferential test of the independence of two nominal variables (*Wolbachia* and MAYV). All analyses were performed by using Prism V 7.4 (GraphPad).

The CLC Microbial Genomics Module (Qiagen, Hilden, Germany) software was used to evaluate the microbiome, both α- and β-diversity parameters. α-diversity was calculated using the Simpson index, which considers richness, uniformity of samples and the 100 most abundant OTUs. The β-diversity analysis was used to compare the distances of the sequenced samples from each other in the intestinal microbiota composition. This analysis can estimate the differences of OTUs between the study samples. To estimate the differences, the UniFrac measure and Principal Coordinate Analysis (PCoA) were used for analysis visualization. To complete the β-diversity data, an analysis was performed using the CLC Microbial Genomics Module software. A phylogenetic OTU tree was constructed using the maximum likelihood method, which is based on the multiple sequence alignment by the MUSCLE (MUltiple Sequence Comparison and Log Expectation) software v.10.1.2.

## 3. Results

**Figure 1:**
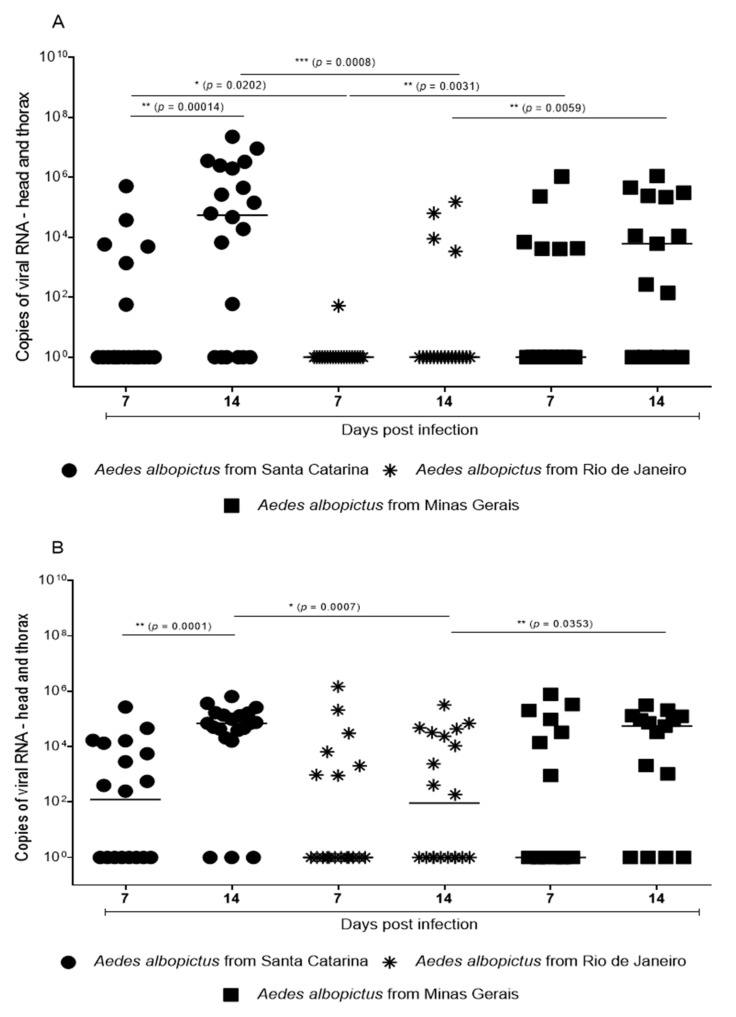
Viral infection rate. Each geometric format represents a single adult mosquito (f). Horizontal lines in black indicate the median absolute copy number of the Mayaro virus in each replicate group tested (**A**,**B**). The asterisks at the top represent the significance after analysis through the Mann–Whitney U-test (* *p* ≤ 0.05; ** *p* ≤ 0.01; *** *p* ≤ 0.001). This figure was generated from the methodology item: Section 2.3.

Our results showed significant effects on susceptibility/infection of the MAYV in all three *Ae. albopictus* populations for 7 and 14 dpi (Figure 1). There was a statistically higher dissemination at 14 dpi when compared to 7 dpi only in *Ae. albopictus* from Santa Catarina (Fisher’s exact test *p* = 0.0013, odds ratio 0.2, 95% CI, 0.1599–0.603).

The population from Santa Catarina showed the highest infection rate (58.7%), followed by the population of Minas Gerais (56.6%) and Rio de Janeiro (28.2%). According to these results, we compared the proportion of infection among the groups, and the population from Rio de Janeiro was different than that from Santa Catarina (Fisher’s exact test *p* = 0.0002, odds ratio 0.2818, 95% CI, 0.3520–0.7511) and Minas Gerais (Fisher’s exact test *p* = 0.0009, odds ratio 0.3004, 95% CI, 0.4023–0.8225). 

**Figure 2:**
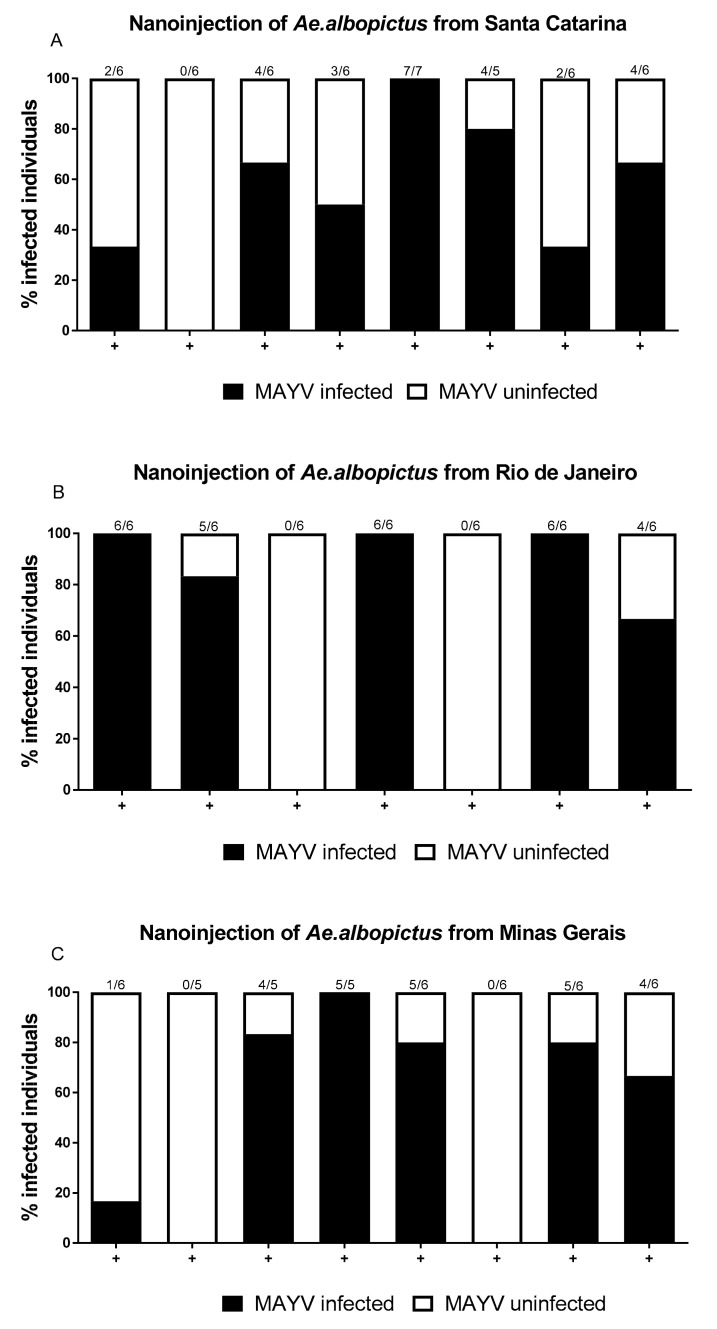
Nanoinjection of different infected saliva groups into naïve *Aedes aegypti* mosquitoes. The saliva was collected from orally infected *Ae. albopictus* with MAYV from Santa Catarina (**A**), Rio de Janeiro (**B**), and Minas Gerais (**C**) mosquitoes, followed by nanoinjection into *Ae. aegypti* naïve mosquitoes. Infected mosquitoes are shown in black and uninfected are depicted in white. Each column represents a single injected-saliva sample from positive head + thorax (+). Each column represents a single saliva sample, and the number of transmission rate mosquitoes nanoinjected is given at the top of each bar. This figure was generated from the methodology item: Section 2.4 and Section 2.5.

The median (viral copies) detected in the head + thorax samples from the first replicate (Figure 1A) were 5.45 × 10^4^ for the Santa Catarina, 5.52 × 10^4^ for the Rio de Janeiro, and 6.12 × 10^3^ for the Minas Gerais samples at 14 dpi. For the second replicate (Figure 2B), the median viral copies for Santa Catarina mosquitoes were 1.24  ×  10^2^ on 7 dpi and 6.88  ×  10^4^ on 14 dpi, 9.2  ×  10^1^ for Rio de Janeiro mosquitoes on 14 dpi, and 5.52  ×  10^4^ copies/head  +  thorax for Minas Gerais mosquitoes on 14 dpi. 

To verify the occurrence of the MAYV in *Ae. albopictus* mosquito saliva, we added a virus amplification step in live naïve *Ae. aegypti*. The MAYV was detected in 26 out of 48 mosquitoes (54.1%) that received saliva from mosquitoes from Santa Catarina (Figure 2A). When saliva originated from Rio de Janeiro mosquitoes, 27 out of 42 injected mosquitoes (64.2%) became infected (Figure 2B). Saliva originating from mosquitoes from Minas Gerais was also able to infect most naïve mosquitoes (24/45; 53.3%) (Figure 2C). Overall, 70% of all the injected saliva samples, irrespective of origin and nanoinjected mosquitoes, were able to infect *Ae. aegypti* mosquitoes through nanoinjection, and therefore proved to be infectious. The rate of infection/transmission of the MAYV, according to dpi, in each population and for both replicates are shown in Table 1.

**Figure 3:**
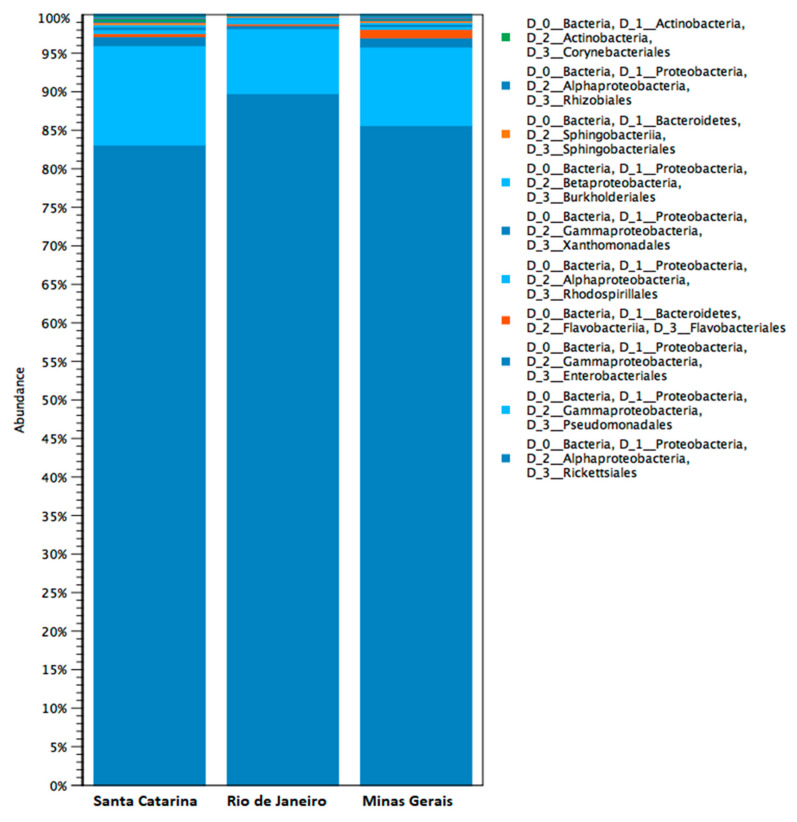
Bar graph generated by the analysis of relative abundance at Order level. The vertical axis shows the relative abundance of each bacterial Order, while the horizontal axis shows the locations of the collected mosquitoes. This figure was generated from the methodology item: Section 2.10.

Regarding the microbiome analysis, 118 OTUs were obtained from the three populations; however, the microbiota of mosquitoes was quite similar between the three groups, and a low diversity of bacteria was observed at all levels of taxonomic classification. The most predominant bacteria belonged to the order *Ricketesiales*, with frequencies higher than 80% in all samples. This order is represented mainly by the genus *Wolbachia*. The results indicated that the locality did not influence the richness of the microbiota and the presence of *Wolbachia* (Supplementary Table S2). Most mosquitoes presented with the genus *Wolbachia*, followed by the genera *Pseudomonas* and *Acinetobacter* (Figure 3).

**Figure 4:**
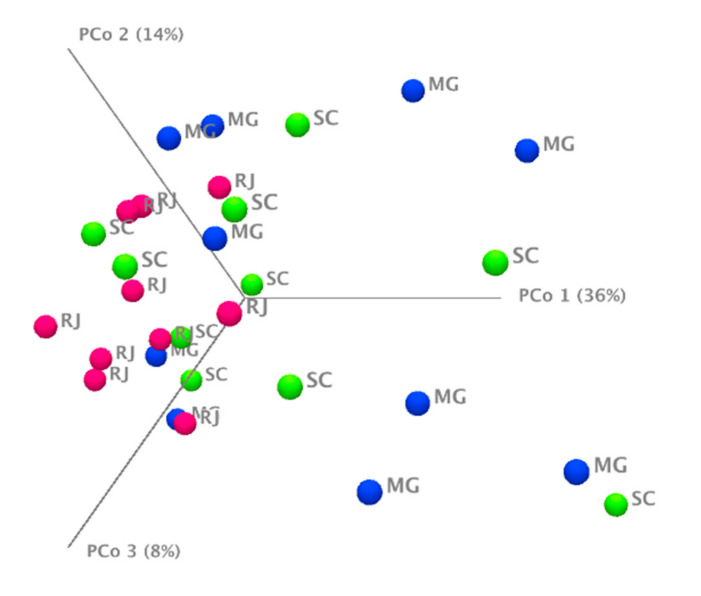
Beta diversity analysis by location. MG: Minas Gerais (blue), SC: Santa Catarina (green), and RJ: Rio de Janeiro (pink). This figure was generated from the methodology item: Section 2.10.

Analyses comparing different parameters, such as region and infection/non-infection of mosquitoes, did not show significant differences between groups. In addition, α- and β-diversity analyses were performed that measured the quantity and abundance of species in homogeneous habitats or different habitats. The sequencing coverage was estimated using rarefaction curves, which starts at approximately 300 reads, indicating that the sequencing was performed at a level good enough to estimate the microbial diversity of the analyzed samples. In the β-diversity analysis, the sample groups were not distant from each other, so the formation of clusters was not observed (Figure 4). To complement the β-diversity data, a PERMANOVA analysis was performed, enabling to compare two groups at a time. The significant difference between the groups of samples analyzed was not confirmed, and this is likely due to the predominance of the *Wolbachia* bacteria in the microbiota of the mosquitoes.

**Figure 5:**
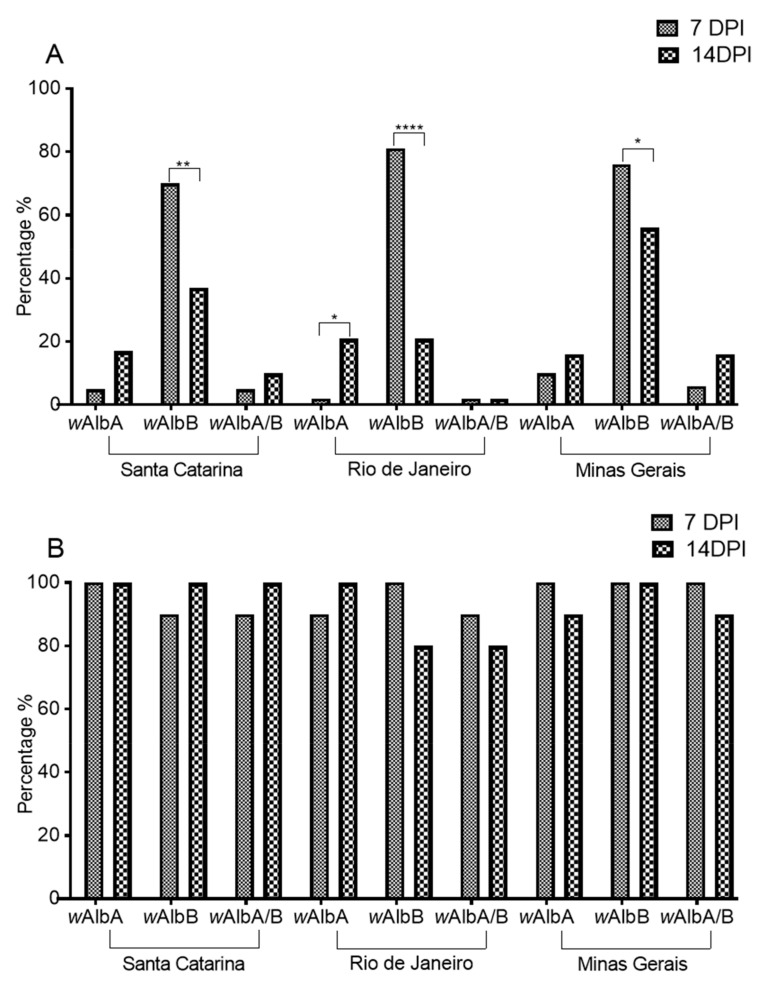
Presence of *Wolbachia* strains (*w*AlbB, *w*AlbB, and *w*AlbA/B) in *Aedes albopictus* mosquitoes at 7 and 14 dpi after Mayaro virus infection—(**A**) head + thorax samples and (**B**) abdomen samples. **A**—The asterisks at the top represent the significance after analysis through the Mann–Whitney U-test (* *p* ≤ 0.05; ** *p* ≤ 0.01; **** *p* ≤ 0.0001). **B**—No statistical difference (Fisher’s exact test). This figure was generated from the methodology item: Section 2.11.

The presence of *Wolbachia* (*w*AlbA and *w*AlbB strains) in mosquitoes (in head + thorax) exhibited higher density at 7 dpi in comparison to 14 dpi (Figure 5A). All populations were statistically different (Fisher’s exact test) between 7 and 14 dpi—Santa Catarina *p* = 0.0067, odds ratio 3.889, 95% CI, 1.5320–9.870; Rio de Janeiro *p* < 0.0001, odds ratio 16.07, 95% CI, 5.171–49.95; and Minas Gerais *p* = 0.0169, odds ratio 4.297, 95% CI, 1.412–13.07.

Overall, the *w*AlbA strain was less present than the *w*AlbB strain. For the Rio de Janeiro population, its titer exhibited an opposite scenario in relation to the *w*AlbB strain, when titers increased at 14 dpi (Fisher’s exact test *p* = 0.0287, odds ratio 0.1071, 95% CI, 0.01266–0.9096). Regarding the presence of both the *w*AlbA and *w*AlbB strains in the same mosquito, this was more consistent at 14 dpi for mosquitoes from Santa Catarina and Minas Gerais.

The presence of *Wolbachia* (*w*AlbA and *w*AlbB strains) in the abdomens of mosquitoes was greater than 97% in all three populations, as well as the presence of *w*AlbA and *w*AlbB strains in the same mosquito (Figure 5B). The population was not statistically different (Fisher’s exact test) between 7 and 14 dpi.

**Figure 6:**
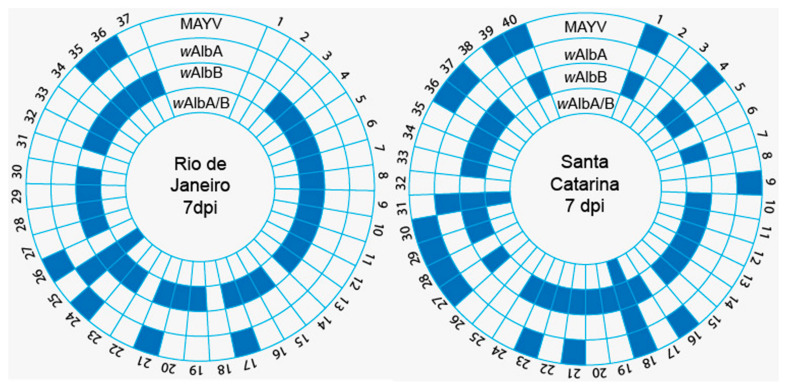
Relationship between Mayaro virus and *Wolbachia* strains in the *Aedes albopictus* at 7 dpi. Positive mosquito sample (blue) and negative (white). This figure was generated from the methodology item: Section 2.11.

When correlating the presence of *Wolbachia* with the MAYV infection rate, we observed that there was dependence between the *w*AlbB strain at 7 dpi for Rio de Janeiro (Pearson *x*^2^ = 0.0237; Pr = 0878) and Santa Catarina (Pearson *x*^2^ = 0.1270; Pr = 0722) populations, meaning that the greater the presence of this strain is, the lower the mosquito infection rate (Figure 6).

## 4. Discussion

The emergence and re-emergence of arboviruses, such as Zika virus (ZIKV), yellow fever, and CHIKV has leveraged the number of studies focusing on understanding the whole of disease vectors, such as *Ae. aegypti* and *Ae. albopictus*, in the transmission potential for these viruses. To date, many authors have suggested that *Ae. albopictus* may play a role in the transmission of these distinct pathogens worldwide [10,11]. However, little is known about the susceptibility status of this mosquito species for the MAYV.

Our results confirm that three geographically distinct *Ae. albopictus* populations from Brazil are susceptible to MAYV infection in the laboratory. Among the three populations, mosquitoes from Rio de Janeiro displayed lower rates of disseminated infection (around 50% less) in comparison to mosquitoes from the other two localities.

There are several potential reasons for this low rate of disseminated infection in the population of *Ae. albopictus* from Rio de Janeiro, including: (1) the anatomic barriers of the insect can determine the efficiency of the infection in the vector [40]; (2) the presence of symbiotic bacteria bearing antiviral genes [41] that can stimulate the mosquito’s immune system or that compete for resources with the virus; (3) inducing immune response [12], either by bacteria or viruses; and (4) the geographical origin of the mosquito may also affect its vector competence, since different genotype/genotype interactions between the virus and vector may occur [42,43,44].

It is worth noting that sporadic/frequent contact between the vector and virus might induce a change in overall vector competence, as distinct studies have shown that the re-emergence of CHIKV (another arbovirus from the *Togaviridae* family) may have been facilitated by the genetic adaptation of the virus to the *Ae. albopictus* vector [45,46].

In both our replicates, it was possible to observe that Santa Catarina mosquitoes had a median viral load of 10^4^ on 14 dpi, whereas it was 10^3^ for Minas Gerais and 10^1^ copies/head + thorax for Rio de Janeiro mosquitoes. However, it is noteworthy that, when analyzed at the individual level, many mosquitoes had a median infection intensity above 10^5^ viral copies, depicting a relevant susceptibility/interaction with the MAYV. 

There is some evidence/speculation that *Ae. albopictus* have a satisfactory infection/dissemination and a large transmission capacity for the MAYV [36,47,48,49]. Wiggins et al. 2018 [47] observed in an oral infection assay that *Ae. albopictus* mosquitoes had a significantly higher infection rate when compared to that of *Ae. aegypti* mosquitoes. Their results are like those observed for our populations from Santa Catarina and Minas Gerais.

Diop et al. 2019 [12] observed increased expression levels of the type C1 gene (NPC1-gene responsible for facilitating the infection of mosquitoes when infected with dengue) in infected *Ae. albopictus* mosquitoes, but not in *Ae. aegypti* mosquitoes. The differences in these levels of gene expression during MAYV infection could explain the variation in vector competence. We have tested the expressions of NPC1 and ML26A genes for *Ae. albopictus* and *Ae. aegypti* (control group); however, the expression of these genes was insignificant when compared to Diop et al. 2019 [12]. One possible explanation for this difference was the age of the mosquitoes. Diop et al. 2019 [12] used 3 dpi mosquitoes, while we used 7 dpi mosquitoes in our analysis.

To the best of our knowledge, there are few studies addressing the vector competence of Brazilian *Ae. albopictus* mosquitoes for the MAYV [50,51]. Smith and Francy 1991 [50] showed that, when these mosquitoes were fed viraemic hamster blood, the infection frequency ranged from 9% to 16%. The authors concluded that their mosquito population was mostly refractory to MAYV infection. Already, in a more recent study, Pereira et al. 2020 [51] suggested that these mosquitoes could play a significant role in the transmission of the MAYV, since this species showed significant vector competence for the MAYV under laboratory conditions. In our case, our results are in line with the latest study.

Smith and Francy 1991 [50] noted in their study that approximately half of the mosquitoes infected with viraemic hamster blood were able to transmit the MAYV when their saliva was tested in capillary tubes. Their results were very similar to our results when we used mosquitos from Rio de Janeiro, where we observed a low virus dissemination rate but a high transmission for the MAYV (over 50%). On the other hand, transmission results for populations from Santa Catarina and Minas Gerais were like Wiggins et al. 2018 [47] (38–76%) and Pereira et al. 2020 [51] (71.1%).

The bacterial microbiota analysis showed *Wolbachia* as the most abundant bacteria for all *Ae. albopictus* populations, constituting >80% bacterial abundance. Following this analysis, using diagnostic primers for *Wolbachia*, two strains of *Wolbachia* (*w*AlbA and *w*AlbB) were detected. Wang et al. 2018 [52] observed that *Wolbachia* was extremely common in adult *Ae. albopictus* mosquitoes, constituting >50% bacterial abundance, with the presence of both strains. This bacterium can cause some alterations in *Ae. albopictus* mosquitoes, such as reduced mosquito lifespan, cytoplasmic incompatibility, and vector competence to viruses [53,54]. 

Rossi et al. 2015 [55] described a mutual exclusion between *Wolbachia* and *Asaia* in *Aedes* and *Anopheles* mosquitoes, which may explain why our mosquito samples exhibited a low abundance of *Asaia* (2 out of 30 samples) and very high abundance of *Wolbachia* (all 30 samples). Consistent with our results, the same pattern was observed by Wang et al. 2018 [52].

Besides *Wolbachia*, other bacteria such as *Chromobacteria, Proteus* or *Paenibacillus* have been related with the inhibition or enhancement of viruses [56,57]. However, in our study, in addition to *Wolbachia*, only *Proteus* was found, with the latter also being known to inhibit DENV-2 when administered to mosquitoes in the blood meal [58].

Consistent with our results, the high prevalence of *Acinetobacter* has been previously reported in *Ae. Albopictus* [59], as being involved in blood digestion. *Enterobacter* and *Acinetobacter* act as an attractant to gravid females and are known to play important roles in the parasite–vector interaction [60].

When correlating the presence of *Wolbachia* with the infection rate of the MAYV, we observed that there was a correlation between the *w*AlbB strain at 14 dpi for Santa Catarina and Rio de Janeiro mosquitoes (i.e., the greater the presence of this strain is, the lower the mosquito infection rate). The blocking effect of *Wolbachia w*AlbB strain seems to depend on the interaction between the host and pathogen. Studies have shown that the *w*AlbB strain in *A. gambiae* inhibits *Plasmodium falciparum*, but for *P. berghei*, the infection is exacerbated [61]. The relationship between the *w*AlbB strain and the *Culex tarsalis* mosquito increases infection with the West Nile virus [62] and blocks the transmission of dengue [56] and CHIKV [63]. 

In *Ae. aegypti* mosquitoes, the introduction of the *w*AlbB strain reduces the transmission of dengue and ZIKV [64], and it has been observed that this strain is more heat stable, indicating that it may be a promising tool in countries with elevated temperatures [65].

Although no correlation was observed in our experiments with the *w*AlbA strain and MAYV infection, it is known that, when this strain was introduced into *Ae. aegypti* mosquitoes, it had higher tissue distribution and higher densities in somatic tissues compared to those in the *w*AlbB strain, suggesting that this strain may strongly inhibit viruses [66]. Studies have shown the viral blocking capacity of the *w*AlbA strain in *Ae. aegypti* mosquitoes, both via intrathoracic nanoinjection of ZIKV and dengue and oral feeding with ZIKV [42]. For the Semliki Forest virus, through thoracic injection, no reduction in copies of the viral genome was detected compared to controls without *Wolbachia* [66].

One possible hypothesis for the higher presence of the *w*AlbB strain at 7 dpi and low presence at 14 dpi can be correlated to a number of factors such as lipids, as neither *Wolbachia* nor the virus have the necessary machinery to produce this nutrient, therefore, depending on the host [67], virus and *w*AlbB competition, membrane synthesis, and nucleic acid production pathways, for example. The *Wolbachia* mechanisms are not fully understood, the role of *Wolbachia*-induced innate immunity priming in pathogen interference is still an object of debate however clearer picture starts to emerge. Some hypotheses suggest that *Wolbachia* depend on host nutrients such as amino acids and lipids [68,69,70], thus being able to create a competition with viruses for subverting the same resources. 

In *Ae. aegypti* mosquitoes, studies of the *w*AlbB strain showed that this bacterium was able to block dengue as effectively as the *w*Mel strain, and the characterization of life history traits showed the *w*AlbB strain resulted in minimal host fitness costs [71,72]. Flores et al. 2020 [73] measured the susceptibility to dengue infection of *Ae. aegypti* lines infected with different *Wolbachia* strain. For the *w*AlbB strain, only a blockade of intermediate degree was observed, such as that seen with abdominal infections in the oral feeding experiments. Mechanistically, the authors suggest that this may indicate differences in efficacy between strains and tissue type. However, it is difficult to compare studies, since the *Wolbachia* densities and tropism may underlie the observed differences.

Studies on different *Wolbachia* strains can direct and add to the list the strains that have potential as tools for use in arbovirus control, so it is important that we understand the relationship that transinfections (technique that allows the insertion of a strain into an unnatural host) may exert on different hosts.

The interaction of the MAYV and *Ae. albopictus* suggests a potential risk of this vector to public health. This becomes even more relevant given the detection of MAYV-positive sera in animals from a wide variety of taxa [17,74], combined with the fact that *Ae. albopictus* are known to be opportunistic mosquitoes, displaying a broad range of hosts on which it feeds, making this vector a potential bridge by which spillover events can occur.

Together, our data show that, regardless of the population, *Ae. albopictus* mosquitoes from distinct regions of Brazil have high susceptibility and vector competence for the MAYV, which may ultimately contribute to the persistence and spread of this specific virus in the field. Finally, we need to stress that these experiments were performed under optimal controlled laboratory conditions; as such, the exact contribution of this mosquito species in the transmission of the MAYV in the field remains to be confirmed, as well as the real contribution of the *w*AlbA/B strain in blocking the MAYV.

## Figures and Tables

**Table 1:** *Aedes albopictus* dissemination and transmission rates for Mayaro virus in two independent experiments.

Blood Meal Titers (PFU/mL)	*Aedes albopictus* Population	Head + Thorax Dissemination Rate (%)		Transmission Rate (Based on Saliva)
1 × 10^9^	Santa Catarina	7 *	30%	Santa Catarina 54.1%
		14 *	70%	
	Rio de Janeiro	7	5%	
		14	20%	
	Minas Gerais	7	40%	Rio de Janeiro 64.2%
		14	66.6%	
6 × 10^9^	Santa Catarina	7	50%	
		14	85%	
	Rio de Janeiro	7	36.8%	Minas Gerais 53.3%
		14	50%	
	Minas Gerais	7	46.6%	
		14	73.3%	

* 7 and 14 represent the days post viral infection. Initial viral titer was determined by plaque formation assay. This Table was generated from the methodology item: Section 2.3, Section 2.4 and Section 2.5.

## Data Availability

The data presented in this study are available in the article and supplementary material.

## Supplementary Materials

The following are available online at https://www.mdpi.com/article/10.3390/pathogens10050525/s1, Table S1: Primers used in the metagenomics assay, Table S2: Statistical analyses of the *Aedes* sp. Microbiome from different locality-PERMANOVA analysis (D_0 UniFrac).
