# Supplementary material for: Mayaro Virus: The Potential Role of Microbiota and Wolbachia
Source: Pathogens. 2021 Apr 27;10(5):525. doi: 10.3390/pathogens10050525 (PMC8145793; doi:10.3390/pathogens10050525)
Supplement: Supplementary file 1 [file pathogens-10-00525-s001.zip › pathogens-1134069-supplementary.pdf]

Supplementary Table 1. Primers used in the metagenomics assay.

Primer	Adapter	Index	Estabilizer	Especific 16S rRNA
0	CAAGCAGAAGACGGCATACGAGAT	TCCCTTGTCTCC	AGTCAGTCAGCC	GGACTACHVGGGTWTCTAAT
1	CAAGCAGAAGACGGCATACGAGAT	ACGAGACTGATT	AGTCAGTCAGCC	GGACTACHVGGGTWTCTAAT
2	CAAGCAGAAGACGGCATACGAGAT	GCTGTACGGATT	AGTCAGTCAGCC	GGACTACHVGGGTWTCTAAT
3	CAAGCAGAAGACGGCATACGAGAT	ATCACCAGGTGT	AGTCAGTCAGCC	GGACTACHVGGGTWTCTAAT
4	CAAGCAGAAGACGGCATACGAGAT	TGGTCAACGATA	AGTCAGTCAGCC	GGACTACHVGGGTWTCTAAT
5	CAAGCAGAAGACGGCATACGAGAT	ATCGCACAGTAA	AGTCAGTCAGCC	GGACTACHVGGGTWTCTAAT
6	CAAGCAGAAGACGGCATACGAGAT	GTCGTGTAGCCT	AGTCAGTCAGCC	GGACTACHVGGGTWTCTAAT
7	CAAGCAGAAGACGGCATACGAGAT	AGCGGAGGTTAG	AGTCAGTCAGCC	GGACTACHVGGGTWTCTAAT
8	CAAGCAGAAGACGGCATACGAGAT	ATCCTTTGGTTC	AGTCAGTCAGCC	GGACTACHVGGGTWTCTAAT
9	CAAGCAGAAGACGGCATACGAGAT	TACAGCGCATAC	AGTCAGTCAGCC	GGACTACHVGGGTWTCTAAT
10	CAAGCAGAAGACGGCATACGAGAT	ACCGGTATGTAC	AGTCAGTCAGCC	GGACTACHVGGGTWTCTAAT
11	CAAGCAGAAGACGGCATACGAGAT	AATTGTGTCGGA	AGTCAGTCAGCC	GGACTACHVGGGTWTCTAAT
12	CAAGCAGAAGACGGCATACGAGAT	TGCATACACTGG	AGTCAGTCAGCC	GGACTACHVGGGTWTCTAAT
13	CAAGCAGAAGACGGCATACGAGAT	AGTCGAACGAGG	AGTCAGTCAGCC	GGACTACHVGGGTWTCTAAT
14	CAAGCAGAAGACGGCATACGAGAT	ACCAGTGACTCA	AGTCAGTCAGCC	GGACTACHVGGGTWTCTAAT
15	CAAGCAGAAGACGGCATACGAGAT	GAATACCAAGTC	AGTCAGTCAGCC	GGACTACHVGGGTWTCTAAT
16	CAAGCAGAAGACGGCATACGAGAT	GTAGATCGTGTA	AGTCAGTCAGCC	GGACTACHVGGGTWTCTAAT
17	CAAGCAGAAGACGGCATACGAGAT	TAACGTGTGTGC	AGTCAGTCAGCC	GGACTACHVGGGTWTCTAAT
18	CAAGCAGAAGACGGCATACGAGAT	CATTATGGCGTG	AGTCAGTCAGCC	GGACTACHVGGGTWTCTAAT
19	CAAGCAGAAGACGGCATACGAGAT	CCAATACGCCTG	AGTCAGTCAGCC	GGACTACHVGGGTWTCTAAT
20	CAAGCAGAAGACGGCATACGAGAT	GATCTGCGATCC	AGTCAGTCAGCC	GGACTACHVGGGTWTCTAAT
21	CAAGCAGAAGACGGCATACGAGAT	CAGCTCATCAGC	AGTCAGTCAGCC	GGACTACHVGGGTWTCTAAT
22	CAAGCAGAAGACGGCATACGAGAT	CAAACAACAGCT	AGTCAGTCAGCC	GGACTACHVGGGTWTCTAAT
23	CAAGCAGAAGACGGCATACGAGAT	GCAACACCATCC	AGTCAGTCAGCC	GGACTACHVGGGTWTCTAAT
24	CAAGCAGAAGACGGCATACGAGAT	GCGATATATCGC	AGTCAGTCAGCC	GGACTACHVGGGTWTCTAAT
25	CAAGCAGAAGACGGCATACGAGAT	CGAGCAATCCTA	AGTCAGTCAGCC	GGACTACHVGGGTWTCTAAT
26	CAAGCAGAAGACGGCATACGAGAT	AGTCGTGCACAT	AGTCAGTCAGCC	GGACTACHVGGGTWTCTAAT
27	CAAGCAGAAGACGGCATACGAGAT	GTATCTGCGCGT	AGTCAGTCAGCC	GGACTACHVGGGTWTCTAAT
28	CAAGCAGAAGACGGCATACGAGAT	CGAGGGAAAGTC	AGTCAGTCAGCC	GGACTACHVGGGTWTCTAAT
29	CAAGCAGAAGACGGCATACGAGAT	CAAATTCGGGAT	AGTCAGTCAGCC	GGACTACHVGGGTWTCTAAT
30	CAAGCAGAAGACGGCATACGAGAT	AGTTACGAGCTA	AGTCAGTCAGCC	GGACTACHVGGGTWTCTAAT

*Primer forward sequence (F) is the same for all samples: (AATGATACGGCGACCACCGAGATCTACACTATGGTAATTGTGTGCCAGCMGCCGCGGTAA).

Supplementary Table 2. Statistical analyzes of the *Aedes* sp. Microbiome from different locality - PERMANOVA analysis (D_0 UniFrac).

Variable	Groups	Pseudo-f statistic	p-value
Locality	SC, RJ and MG	1.54482	0.10573

Group 1	Group 2	Pseudo-f statistic	p-value	p-value (Bonferroni)
SC	RJ	1.43406	0.15376	0.46128
SC	MG	0.72792	0.61701	1.00000
RJ	MG	2.72587	0.02471	0.07413

*SC (Santa Catarina), RJ (Rio de Janeiro) and MG (Minas Gerais).
